# Influence of Weekday and Seasonal Trends on Urgency and In-hospital Mortality of Emergency Department Patients

**DOI:** 10.3389/fpubh.2022.711235

**Published:** 2022-04-25

**Authors:** Jennifer Hitzek, Antje Fischer-Rosinský, Martin Möckel, Stella Linnea Kuhlmann, Anna Slagman

**Affiliations:** ^1^Emergency and Acute Medicine, Charité—Universitätsmedizin Berlin, Berlin, Germany; ^2^Department of Neurology, Charité - Universitätsmedizin Berlin, Berlin, Germany

**Keywords:** utilization (U), emergency department (ED), seasonal trends, temporal trends, urgency, hospital mortality, secondary data analysis

## Abstract

**Background:**

Given the scarcity of resources, the increasing use of emergency departments (ED) represents a major challenge for the care of emergency patients. Current health policy interventions focus on restructuring emergency care with the help of patient re-direction into outpatient treatment structures. A precise analysis of ED utilization, taking into account treatment urgency, is essential for demand-oriented adjustments of emergency care structures.

**Methods:**

Temporal and seasonal trends in the use of EDs were investigated, considering treatment urgency and hospital mortality. Secondary data of 287,119 ED visits between 2015 and 2017 of the two EDs of Charité Universitätsmedizin Berlin, Campus Charité Mitte and Campus Virchow Klinikum were analyzed.

**Result:**

EDs were used significantly more frequently on weekends than on weekdays (Mdn = 290 vs. 245 visits/day; *p* < 0.001). The proportion of less urgent, outpatient emergency visits on weekends was above average. Holiday periods were characterized by at least 6, and at most 176 additional ED visits. In a comparison of different holidays, most ED visits were observed at New Year (+68% above average). In addition, a significant increase in in-hospital mortality on holidays was evident among inpatients admitted to hospital *via* the ED (3.0 vs. 3.2%; *p* < 0.001), with New Year's Day being particularly striking (5.4%).

**Conclusion:**

These results suggest that, in particular, the resource planning of outpatient emergency treatment capacities on weekends and holidays should be adapted to the increased volume of non-urgent visits in EDs. Nevertheless, treatment capacities for the care of urgent, inpatient emergencies should not be disregarded and further research projects are necessary to investigate the causes of increased mortality during holiday periods.

## Introduction

The increasing use of emergency departments (ED) poses a major challenge for the care of emergency patients. In comparison with other countries who are part of the Organization for Economic Co-operation and Development (OECD), Germany has an average annual growth rate of 4.9% in the use of ED visits that result in inpatient treatment. This is significantly above the average (2.4%) of other OECD countries and thus Germany shows the fourth highest growth rate (1). Furthermore, Berlin is the federal state with the highest number of outpatient ED visits, although this number has been declining modestly since 2016 (2–4). The causes for the increasing case numbers might be limited resources of other health care providers (i.e., long waiting times to specialist treatment or elective procedures), but effects of demographic change with increasing numbers of older patients with complex medical and nursing needs are also reported (1, 5). Another factor might be changes in society's attitude toward standards and expectations of comprehensive medical treatment at the highest technical and scientific level, as well as the day-to-day availability of EDs, which seem to lead to a preferred usage of EDs by younger patients with less urgent conditions (6, 7). In addition, structural problems of the outpatient emergency care system and patients' lack of knowledge of treatment alternatives by statutory health insurance (SHI)-accredited physicians are associated with increasing case numbers in EDs (5, 7–9). Emergency care in Germany is provided by three independent sectors, which are organized on a federal basis. The outpatient treatment by physicians in private practice and the on-call service of the Association of Statutory Health Insurance Physicians (SHI), the rescue service, and inpatient care by the emergency departments (10). For adequate emergency treatment in the ED, the availability of room and personnel capacities, diagnostic equipment and, above all, patient number in relation to these resources are decisive for the quality and efficiency of care processes. A reduction in the quality of medical care and effects on medical outcomes, e.g., mortality, have already been demonstrated in studies on ED-crowding (7, 11, 12). Next to the shortage of specialists and the reduction of hospital beds, the increased use of emergency departments by patients, requiring less urgent care, has been identified as a trigger for crowding (7, 11, 12). In addition, negative effects on care and mortality have been discussed in the context of hospital admission on weekends, known as the “weekend-effect (13). An examination of weekday and temporal and seasonal trends in the use of ED with regards to treatment urgency thus provides important insights for a more efficient planning of available resources and alternative treatment options.

In the present study, temporal and seasonal trends of ED utilization with special regard to treatment urgency were examined in secondary data of two urban, tertiary care EDs of the Charité–Universitätsmedizin Berlin over a period of 3 years.

## Materials and Methods

The study included secondary data of 287,119 emergency visits in the EDs of the Charité-Universitätsmedizin Berlin, Germany, Campus Mitte (CCM) and Campus Virchow-Klinikum (CVK) between 2015 and 2017. These are two emergency rooms, of maximum-care hospitals and located in the inner-city area of Berlin. The CCM ED is an interdisciplinary ED with an attached emergency ward. Emergency care at CVK, on the other hand, is organized into three independent EDs. These include a surgical ED, an internal ED with an attached emergency ward, and the pediatric ED. Patients in the pediatric ED and patients who visited one of the EDs due to an accident at work were not included in the analyses.

### Data

As part of the analysis, data collected and stored for quality assurance purposes from the EDs of CCM and CVK were used. In this context, data sets from 2015 to 2017 were extracted electronically from the hospital information system and converted into a table format. Subsequently, the data set was compressed to the aspects relevant to the research question before the data set was transferred to the statistics program SPSS for data preparation and subsequent analysis. Taking into account the underlying research interest, the case level was defined as the unit of analysis. In case that patients visited the ED several times during the study period, the different visits were considered as a separate case in the analysis. This offers the possibility to show a differentiated picture of the actual use of ED.

In the medical context, the term “season” is associated with a higher incidence of certain diseases within a certain period of time, for example, seasonal fluctuations in infectious diseases (14). There is currently no generally valid definition of seasonality in the medical context. In the current study, “seasonal trends” were defined as “cyclical fluctuations that repeat at regular intervals and do not exceed a period of 1 year”: seasons, month, school vacation, and holidays (15). “Temporal trends” were defined as daily fluctuations with reference to the time of day. In-hospital mortality analyses was defined as the event of death either in the ED or during subsequent inpatient stay. Other variables considered in the analyses, as well as their operationalization presented in Table 1. All Data were managed and analyzed using IBM SPPS Statistics V25. Quantitative characteristics were described by median (Mdn), first (Q1) and third quartile (Q3) and maxima and minima. Qualitative variables were described by relative and absolute frequencies. Before performing adequate statistical tests, a visual test for normal distribution was performed using histograms for the variables “number of cases per day” and “age”. In addition to the visual test, the Kolmogorov-Smirnov and Shapiro-Wilk tests were also used to check for normal distribution. For group comparisons the Chi-square test was used for categorical variables and due to skewed distributions Kruskal Wallis tests were performed for quantitative variables. A *p*-value of *p* < 0.05 was considered as significant. Due to the descriptive, exploratory nature of this work, no corrections for multiple testing were performed. The effect of holidays, weekends, school vacations and seasons on ED utilization were analysed by multiple linear regression. Outliers were analyzed and defined by the IQR method of Tukey. The interquartile range (IQR) was calculated by the difference of the third and first quartile (25 and 75 percentiles). We investigated outliers for two different scenarios; 1.5 times the IQR and 3 times the IQR. To compute the lower and upper border of the IQR we subtracted this value form the first quartile and added this value to the third quartile. All residuals with higher or lower values of the calculated 1.5x IQR and 3x IQR were defined as outliers.

**Table 1:** Definition and Operationalization of variables included in analyses.

**Variables**	**Description**	
**Variables with seasonal reference**		
Seasons	Spring, Summer, Autumn, Winter (astronomical classification)	
Month	January, February, March, April, May, June, July, August, September, October, November, December	
School vacations Berlin/Brandenburg	Winter holidays	02.02.−07.02.2015/01.02.−06.02.2016 31.01.−04.02.2017
	Easter holidays	30.03.−11.04.2015/21.03.−02.04.2016 10.04.−22.04.2017
	Whitsun holidays	26.05.2015/17.05.−18.05.2016 06.06.−09.06.2017
	Summer holidays	16.07.−28.08.2015/21.07.−03.09.2016 20.07.−01.09.2017
	Autumn holidays	19.10.−31.10.2015/17.10 −28.10.2016 23.10.−04.11.2017
	Christmas holidays	01.01.−02.01.2015/23.12.−03.01.2017 21.12.−31.12.2017
	Single day holidays	15.05.2015/06.05.2016/24.05.2017 26.05.2017 /02.10.2017
Day of the week	Monday, Tuesday, Wednesday, Thursday, Friday, Saturday, Sunday	
Holidays	New Year	01.01.2015/16/17
	Good Friday	03.04.2015/25.03.2026/14.04.2017
	Easter Sunday	05.04.2015/27.03.2016/16.04.2017
	Easter Monday	06.04.2015/28.03.2016 /17.04.2017
	Ascension Day	14.05.2015/05.05.2016 /25.05.2017
	Labor Day	01.05.2015/16/17
	Whit Sunday	24.05.2015/15.05.2016/04.06.2017
	Whit Monday	25.05.2015/16.05.2016/05.06.2017
	German Unification Day	03.10.2015/16/17
	Reformation Day	31.10.2015/16/17
	Christmas Eve	24.12.2015/16/17
	Christmas Day	25.12.2015/16/17
	Christmas Day	26.12.2025/16/17
	27 December	
	New Year's Eve	31.12.2015/16/17
**Variables with time reference**		
Hours	0–23	
**Outcome**		
Emergency visits	Number of emergency visits	
**Dimensions of stratification**		
Age/sex	0–114/ men, women	
Urgency of treatment	Less urgent	category 4–5 (MTS)^*^
	Urgent	category 1–3 (MTS)^*^
Case type	Outpatient, inpatient	
Inpatient mortality	Proportion of patients deceased in hospital	

* *MTS category describes the urgency of treatment using the Manchester triage system*.

The research project was approved by the institutional review board (EA1/082/18) and the institutional data protection department with reference to §24 and §25 LKG Berlin. Neither patients nor the public were involved in the design, or conduct, or dissemination plans of this research. The dataset generated and analyzed during the current study are available from the corresponding author (Jennifer Hitzek) on reasonable request.

## Results

The study population consisted of 142,954 visits of men (49.8%) and 144,151 visits of women (50.2%), for 14 participants gender was documented as unknown. The mean age was 40 (Mdn) years [28;60]. On average, 258 (Mdn) visits attended the EDs per day [238;284], with a minimum of 161 visits and a maximum of 439 visits per day.

### Seasonal Trends

**Figure 1:**
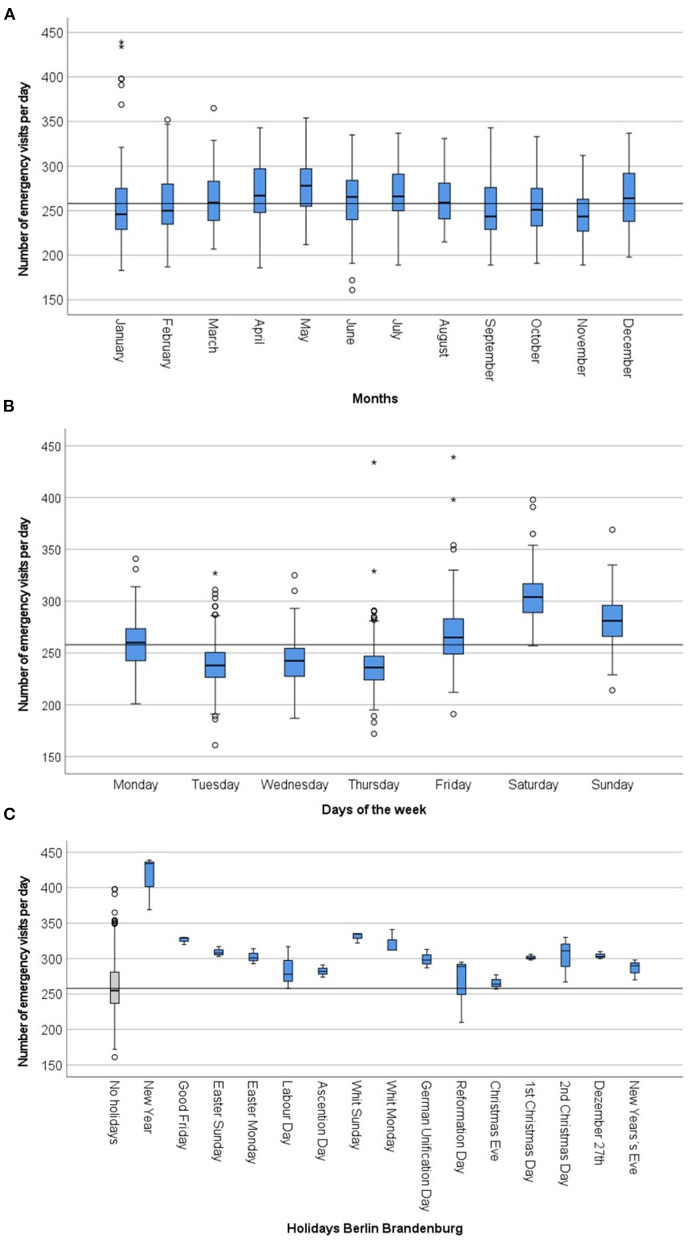
Distribution of emergency visitis per day in comparison of months **(A)** in comparison of weekdays **(B)** and in comparison of holidays **(C)** in the years 2015-2017 in the emergency departments of Charité - Universitätsmedizin Berlin (CVK, CCM). The average daily use of emergency departments (median) in the study population serves as a reference line.

Spring was the season with the highest ED utilization with an average of Mdn = 269 visits per day [250;295]. The fewest visits were registered in autumn, averaging Mdn = 247 visits per day [231;273] (Table 2). With regard to monthly changes, March (Mdn = 259,[239;285]), April (Mdn = 267, [248;298]), May (Mdn = 278, [255;297]), June (Mdn = 266, [240;284]), July (Mdn = 266,[250;291]), August (Mdn = 259, [241;282]) and December (Med = 264, [238;294]) showed above-average patient numbers (Figure 1A; *p* < 0.001), while the months of January (Mdn = 246, [229;276]), February (Mdn = 250, [233;281]), September (Mdn = 244, [229;277]), October (Mdn = 251, [233;276]) and November (Mdn = 243, [238; 294]) showed a below-average utilization.

**Table 2:** Overview of emergency visits by season, day of the week and holiday in total and stratified by demographic characteristics, treatment urgency, case type and mortality in the years 2015–2017 in the emergency departments of Charité - Universitätsmedizin Berlin (CKV, CCM).

		**Utilization behavior**		**Demographics**			**Classification of treatment urgency**		**Case type**		**Inpatient mortality**
		**Number of ED visits,** ** (Mdn,[Q1;Q3])**	**Min/Max**	**ED visits men** ** absolute in (%)**	**ED visits women** ** absolute in (%)**	**age in years,** ** (Mdn, [Q1;Q3])**	**Proportion of less** ** urgent treatment cases** ** absolute in (%)**	**Proportion of urgent** ** treatment cases** ** absolute in (%)**	**Proportion of** ** inpatient** ** treatment** ** cases absolute** ** in (%)**	**Proportion of** ** outpatient** ** treatment** ** cases** ** absolute in (%)**	**Proportion of** ** deceased** ** absolute** ** in (%)**
Seasons	Spring	269, [250;295]	186/365	37,620 (49.9)	37,823 (50.1)	40, [28;59]	33,263 (46.2)	38,779 (53.8)	18,565 (24.6)	56,870 (75.4)	543 (2.9)
	Summer	258, [240;283]	161/337	36,475 (49.7)	36,858 (50.3)	39, [27;58]	32,100 (45.8)	37,943 (54.2)	18,538 (25.3)	54,787 (74.7)	544 (2.9)
	Autumn	247, [231;273]	189/343	33,945 (49.9)	34,051 (50.1)	41, [28;61]	27,958 (43.2)	36,765 (56.8)	18,155 (26.7)	49,829 (73.3)	528 (2.9)
	Winter	253, [235;287]	183/439	34,914 (49.6)	35,419 (50.4)	41, [28;60]	30,672 (45.7)	36,457 (54.3)	17,919 (25.5)	52,400 (74.5)	557 (3.1)
	*p*-value	*p* < 0.001		*p* < 0.812		*p* < 0.001		*p* < 0.001		*p* < 0.001	*p* < 0.001
Weekdays	Monday	260, [242;274]	201/341	20,421 (50.5)	19,983 (49.5)	42, [28;62]	16,635 (43.2)	21,871 (56.8)	11,724 (29.0)	28,670 (71.0)	344 (2.9)
	Tuesday	238, [227;251]	161/327	18,884 (50.5)	18,536 (49.5)	42, [28;62]	15,093 (42.4)	20,505 (57.6)	10,660 (28.5)	26,757 (71.5)	328 (3.1)
	Wednesday	243, [228;255]	187/325	18,958 (50.2)	18,813 (49.8)	42, [28;61]	15,223 (42.4)	20,720 (57.6)	10,320 (27.3)	27,444 (72.7)	338 (3.3)
	Thursday	236, [224;247]	172/434	18,900 (50.7)	18,368 (49.3)	42, [28;62]	14,967 (42.2)	20,498 (57.8)	10,467 (28.1)	26,791 (71.9)	293 (2.8)
	Friday	265, [249;283]	191/439	20,982 (49.8)	21,111 (50.1)	41, [28;60]	18,468 (46.1)	21,608 (53.9)	10,528 (25.0)	31,562 (75.0)	307 (2.9)
	Saturday	304, [289;317]	257/398	23,326 (48.7)	24,573 (51.3)	38, [27;56]	23,158 (50.5)	22,698 (49.5)	9,746 (20.3)	38,151 (79.7)	266 (2.7)
	Sunday	281, [266;296]	214/369	21,483 (48.5)	22,767 (51.4)	38, [27;56]	20,449 (48.1)	22,035 (51.9)	9,732 (22.0)	34,514 (78.0)	296 (3.0)
	*p*-value	*p* < 0.001		*p* < 0.001		*p* < 0.001		*p* < 0.001		*p* < 0.001	*p* < 0.001
Weekdays, Weekends, Holidays	Weekdays	245, [231;262]	161/398	93,858 (50.4)	92,474 (49.6)	42, [28;61]	76,220 (43.0)	101,060 (57.0)	51,958 (27.9)	134,342 (72.1)	1,556 (3.0)
	Weekends	290, [276;311]	214/398	42,261 (48.6)	44,724 (51.4)	38, [27;56]	41,010 (49.2)	42,408 (50.8)	18,413 (21.2)	68,563 (78.8)	525 (2.9)
	Holidays	303, [288;318]	210/439	6,835 (49.6)	6,953 (50.4)	39, [27;57]	6,763 (51.1)	6,467 (48.9)	2,806 (20.4)	10,981 (79.6)	91 (3.2)
	*p*-value	*p* < 0.001		*p* < 0.001		*p* < 0.001		*p* < 0.001		*p* < 0.001	*p* < 0.001
	Totals	**258, [238;284]**	**161/439**	**142,955 (49.8)**	**144,151 (50.2)**	**40, [28;60]**	**123,993 (43.2)**	**149,935 (52.2)**	**73,177 (25.5)**	**213,886 (74.5)**	**2,172 (3.0)**

### Temporal Trends

While the weekdays Tuesday to Thursday were rather under-average, an increase in the number of visits could be shown from Friday onwards, with Saturday being the day with the highest use of the EDs with an average increase of 17.8% as compared to the average ED visits (46 visits more per day) (Figure 1B). On Sundays, the average use was 9.0% higher as compared to the average ED visits (23 visits more per day) (Table 2).

**Figure 2:**
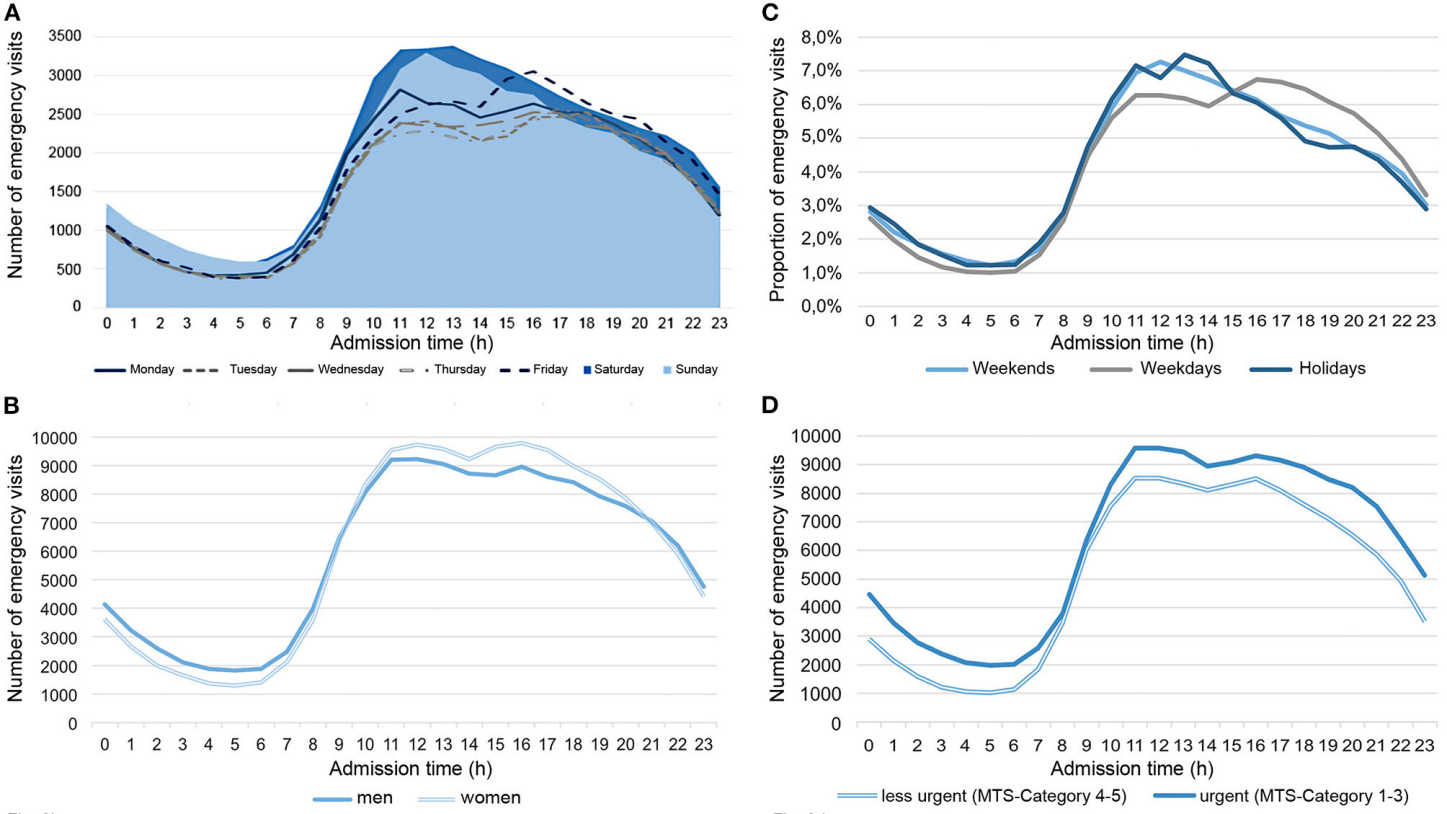
Number of emergency visits depending on admission time in comparison of weekdays **(A)**, gender-specific differences **(B)**, in comparison of weekends, weekdays, holidays **(C)**, and the associated distribution of treatment urgency **(D)** in the years 2015–2017 in the emergency departments of Charité—Universitätsmedizin Berlin (CVK, CCM).

Looking at the daily curve (Figure 2A), a steady increase in the use of EDs from 6:00 a.m. onwards, culminating in the first peak between 10 a.m. and 12 p.m. was found. Afterwards the number of patients in the ED decreased slightly, before another peak occurred between 3 and 5 p.m. An upstream peak on Monday (10 a.m.) was noticeable. On Fridays, however, the peak was shifted to the afternoon (4–5 p.m.).

### ED Utilization During School Vacations and Holidays

In general, EDs were used more frequently during School vacations compared to days outside these periods (Mdn = 265, [246;292] 2 vs. Mdn = 255, [236;282] visits, *p* < 0.001, Table 2). If the annual average use of 258 (Mdn) visits per day [238;284] was taken as a reference, all other School vacations except the autumn and winter holidays showed above-average use of EDs (Table 3). The highest utilization was observed on single day holidays (Mdn = 305, [268;284] and during the Christmas holidays (Mdn = 300, [272;329]).

**Table 3:** Average number of emergency visits compared to holidays in the years 2015 - 2017 in the emergency departments of Charité - Universitätsmedizin Berlin (CVK, CCM).

**School vacations**	**Number of treatment cases**	**Median**	**IQR**	**Min**.	**Max**.
Christmas holidays	11,118	300	272–329	233	439
Winter holidays	4,422	251	242–275	219	347
Easter holidays	11,147	281	254–315	233	365
Whitsun holidays	1,889	272	259–279	257	284
Summer holidays	35,076	259	241–283	215	337
Autumn holidays	9,521	248	236–265	206	311
Single day holidays	1,544	305	268–352	257	354
No holidays	212,402	255	236–282	161	398
Totals	287,119	258	238–284	161	439

EDs were used more frequently on holidays than on non-holidays and in comparison to the annual average (Mdn = 303, [288;319] vs. Mdn = 255, [237;281] vs. Mdn = 258, [238;284] visits per day, *p* < 0.001, Table 2). New Year was characterized by the highest utilization with an average of 154 more visits per day (increase by 68.0%) and a total maximum of 439 visits per day (Figure 1C). On Whit Sunday the increase was 29.0% (77 visits more per day). In contrast to weekdays, ED were increasingly visited in the morning on weekends and holidays (9 a.m. - 1 p.m.). The second peak in ED utilization on weekdays in the afternoon was also not evident here; instead, utilization of ED decreased continuously from 2:00 p.m. onward (Figure 2C).

### Stratification by Age, Gender, Urgency of Treatment, Inpatient Stay and Hospital Mortality

#### Age and Gender Specific ED Utilization

Emergency patients on weekends were on average 4 years younger than patients on weekdays (Mdn = 38, [27;56] vs. Mdn = 42, [28;61]; *p* < 0.001).

The daytime curve showed a gender-specific trend: During the day (10–20 o'clock), more women visited the ED, whereas at night (21–09 o'clock) more men used the ED (Figure 2B).

#### Clinical Characteristics in ED Patients

**Figure 3:**
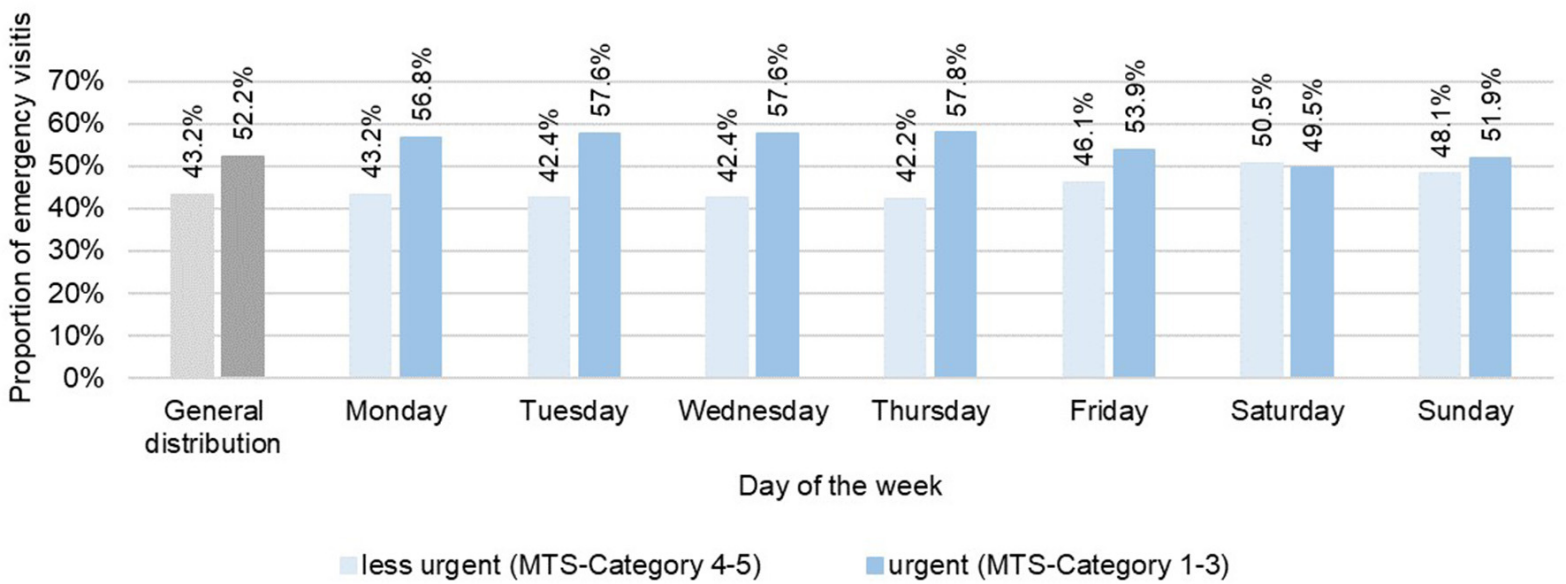
Distribution of treatment urgency in the comparison of weekdays in the years 2015–2017 in the emergency departments of Charité—Universitätsmedizin Berlin (CVK, CCM).

Fifty two percent of the visits were assigned to an urgent treatment category and 43.2% to a less urgent treatment category (Figure 3). The proportion of urgent visits was particularly high between 8 p.m. and 7 a.m. (>55.0%), whereas between 8 a.m. and 7 p.m. treatment urgency was on average (Figure 2D). The ratio of treatment urgency within the weekdays showed clear differences (Table 2): on Monday to Thursday more urgent visits predominated while on Fridays to Sundays the proportion of less urgent visits increased (Figure 3). Similarly, the proportion of urgent visits was lower on all school vacations than on non-vacations (48.9 vs. 55.0%). On 9 of 15 holidays, the proportion of less urgent visits exceeded the proportion of urgent visits. Easter Sunday (57.7%), New Year's Eve (55%) and New Year's Day (54.9%) were characterized by a very high proportion of less urgent ED visits (Table 4).

**Table 4:** Distribution of treatment urgency in comparison of holidays in the years 2015 - 2017 in the emergency departments of Charité - Universitätsmedizin Berlin (CVK, CCM).

	**Treatment urgency less urgent**		**Treatment urgency urgent**	
**Holidays**	**Number of emergency cases**	**Proportion in %**	**Number of emergency cases**	**Proportion in %**
New Year	443	54.9	530	45.1
Good Friday	483	51.2	460	48.8
Easter Sunday	521	57.7	382	42.3
Easter Monday	457	51.3	434	48.7
Ascension Day	437	52.8	391	47.2
Labour Day	370	45.7	439	54.3
Whit Sunday	456	48.8	478	51.2
Whit Monday	493	53.4	430	46.6
German Unification Day	423	49.1	439	50.9
Reformation Day	372	48.4	396	51.6
Christmas Eve	394	51.6	370	48.4
Christmas Day (25.12.)	434	49.4	445	50.6
Christmas Day (26.12.)	445	50.9	430	49.1
27 December	391	44.9	480	55.1
New Year's Eve	443	55.0	363	45.0
Totals	287,119	43.2		52.2

On average, 74.5% of visits resulted in an outpatient treatment and 25.5% in an inpatient treatment, respectively. There were significant differences in the type of admission, both in the comparison of weekdays and holidays. There was a lower proportion of outpatient visits between Monday and Thursday and an increase in outpatient visits from Friday to Saturday (Table 2). Furthermore, the proportion of ED outpatient visits (79.6%) was above the overall average (74.5%) on all holidays and highest on Good Friday and Whit Sunday with 82.5% respectively (Table 2).

**Figure 4:**
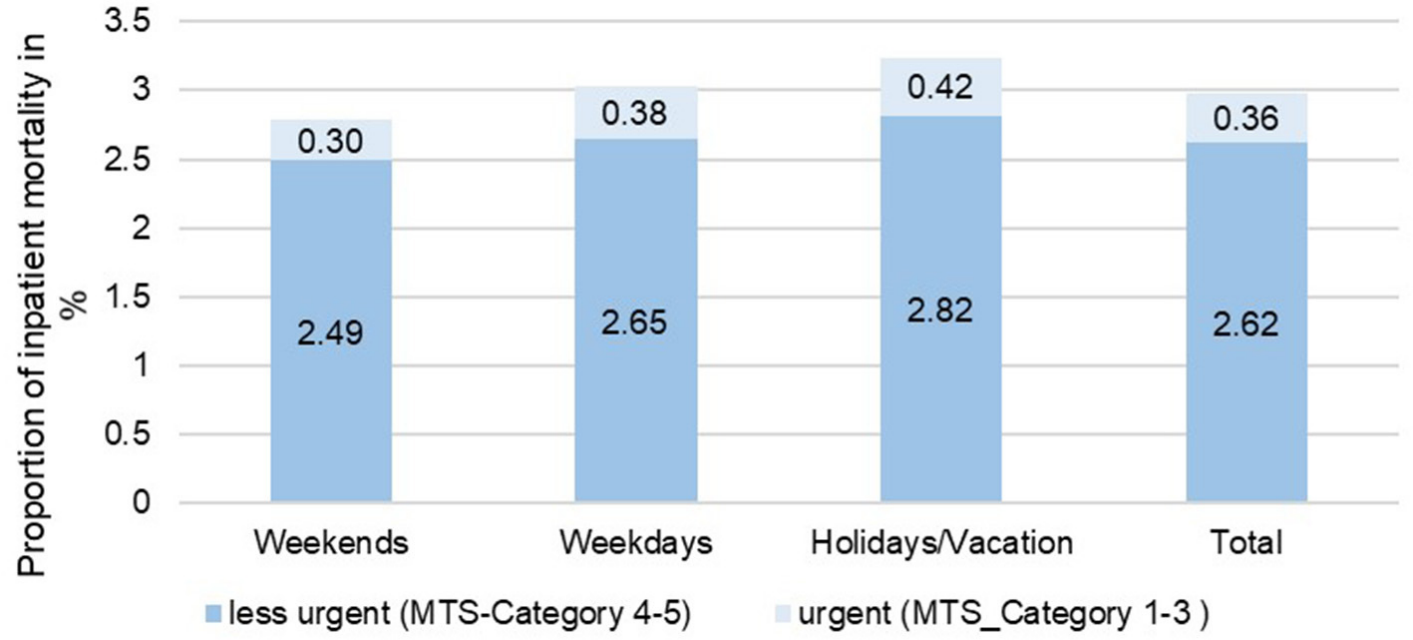
Comparison of hospital mortality of inpatient treatment cases depending on the day of admission, taking into account the urgency of treatment in the years 2015–2017 in the emergency departments of Charité—Universitätsmedizin Berlin (CVK, CCM).

Hospital mortality among inpatients was 3.0%, with an average increase up to 3.2% during holidays (Figure 4). A particularly high in-hospital mortality was observed on New Year's Eve (5.4%), Christmas Day (5.2%), German Unification Day and Christmas Eve (4.7% each). Although there was no increase in average in-hospital mortality when comparing holidays, the Pentecost holidays were characterized by an increase in in-hospital mortality up to 4.2%. In a comparison of the seasons and days of the week (Table 2) only slight differences in in-hospital mortality could be observed.

### Multifactorial Analysis of Effects Between ED Visits and Season, Weekend, Holidays and School Vacation

In the multifactorial linear regression model with the factors season, weekend, holiday and school vacation, a goodness of fit of 0.46 (adjusted R-squared) was achieved. A significant effect was found for all four factors (Table 5, *p* < 0.001). Weekends, holidays and school vacations were found to be factors with a positive effect i.e., increase in ED visits. Summer, autumn and winter, on the other hand, showed a negative effect compared to spring and were associated with a small decrease in ED visits. The predicted values of the model represented outliers in 2.4% (1.5x IQR) and 0.3% (3x IQR) of the cases (first quantile = −16, third quantile = 14). 0.9% of the 2.4% outliers were cases where the model overestimated the utilization of the ED and in 1.5% of the cases fewer ED visits were predicted than were actually observed. For the 3x IQR criterion 0.3% of the values deviated from the predicted model, with four outliers underestimated the actual utilization of the ED.

**Table 5:** Multivariate effects of season, weekends, holidays and school vacations on number of ED visits in the years 2015–2017 in the emergency departments of Charité–Universitätsmedizin Berlin (CCM/CVK).

	**Unstandardized coefficient B**	**95% Confidence interval for B**	**Significance**
Constant (Number of ED visits)	257	(253, 260)	*p* < 0.001
Summer	−18	(−22, −13)	*p* < 0.001
Autumn	−21	(−25, −16)	*p* < 0.001
Winter	−11	(−15, −6)	*p* < 0.001
Weekend	45	(42, 48)	*p* < 0.001
Holidays	32	(25, 40)	*p* < 0.001
School vacations	14	(10, 17)	*p* < 0.001

*1,096 days were investigated in this model. On average 262 (mean) visits attend the ED per day with a standard error of 1.04. A goodness of fit of 0.46 (adjusted R-square) was achieved and the residual standard error was 25.1*.

## Discussion

The current study examined relations between visits to ED at two German hospitals from 2015 to 2017 versus season, weekday, school vacation and holiday with stratification by five groups: age, sex, treatment urgency, case type (inpatient, outpatient), and in-hospital mortality. We found that weekends, school vacations and holidays are associated with an increased number of patients in the ED. The proportion of less urgent, outpatient emergency patients on these days is above average. The hospital mortality of inpatient emergency patients shows hardly any difference when comparing it with the days of the week, but it is above average on holidays and during the Whitsun holidays. Even though only a small number of patients visit the ED in autumn, these patients show the highest urgency of treatment and the highest proportion of inpatients regarding seasonal comparison. In the multifactorial linear regression model, a statistically significant influence could be demonstrated for the factors season, weekend, holidays and school vacation, independently of each other. Weekends and holidays are particularly associated with an increase in emergency department visits.

### Aspects of Seasonal ED Utilization

The analyses of seasons and months could not prove a previously suspected decrease in the number of visits during the summer period. Autumn was characterized by the lowest utilization of EDs, while treatment urgency was higher during this period. Since processes might be prolonged in more urgent visits and treatment times in the ED might thus be longer, this could be one explanation of the perceived crowding of EDs during autumn. These results implicate that the focus of personnel planning should not only be limited to case numbers, but also consider treatment urgency. A month-to-month comparison of the use of EDs showed an increase in April, May and July in particular. Fewer emergency patients visited the ED in January, February, September, October, and November. One possible explanation could be that recreational activities with a high risk of injury are performed more frequently during the months of higher utilization. Sports activities such as bicycling as well as the motorcycle season, are associated with an increased risk of injury, which could be reflected in the increased ED utilization during these months. The highest admission of accident patients to EDs in July has already been demonstrated in a study by Rising et al. (16). In addition, the period between April and July includes a large number of holidays, so that EDs are used more often than average, not only due to increased leisure activities, but also due to an increased number of tourists.

### Holidays, School Vacations and Treatment Urgency

In the context of vacations, EDs offer a quick and low-barrier option for clarifying medical health problems. EDs are used particularly frequently during Christmas holidays and days off from school. The previous statements apply to the same extent for the days without school. The increase in the number of cases during the Christmas holidays is possibly due to the closing times of the outpatient health care system. A large number of general practitioners and specialists closed their practices during the Christmas period, so that the options for medical care are very limited. The increased use of EDs during holidays and on weekends, especially by non-urgent visits could partly be explained by closing times of the outpatient health care system and the lack of knowledge of the population about the outpatient emergency system by the Association of SHI-physicians (3–6). Further research is required to shed light in other causative factors for these observations. It is questionable but still possible that the results can partly be caused by an actual increase in medical emergencies. The increase of outpatient emergencies, up to over 80%, with less urgent treatment needs during holidays and on weekends is consistent with the results of other studies (3, 4, 17). This might be caused by the perceived need of rapid diagnostic clarification and treatment at the highest medical level of care by patients, which is likely to be taken for granted by patients in the ED (6, 8, 18). This advantage is particularly appealing for working people, since appointments in the outpatient care system, especially for specialist treatments, are sometimes associated with long waiting times or cannot be reconciled within working hours.

### In-hospital Mortality and Weekend Effect

Taking into account the results on in-hospital mortality, an interesting area of conflict emerges: Although emergency patients are more likely to have a lower treatment urgency during holidays and the proportion of outpatients is higher (4, 19), in-hospital mortality of inpatients is increased during holidays. A reduction in quality of medical care and a negative impact on outcomes such as mortality have already been demonstrated in the context of crowding in EDs (11, 12) and are underscored by the results presented in this paper. Internationally discussed negative effects on mortality at weekends could not be proven in the current analyses, this is in line with finding suggesting that the weekend effect is less pronounced in ED-patients (13). In fact, this may be a selection bias of university hospitals, as they are usually staffed 24/7 with specialist physicians in contrast to non-university hospitals. As a result, specialist treatment was available at all times in the investigated study population, so there are fewer delays in treatment and diagnosis than might be observed in other hospitals at weekends.

### Aspects of Temporal ED Utilization and Practical Implications

The temporal trends regarding time of admission to the ED are in line with previous studies and might be helpful for planning of alternative resources (6, 20). In addition, the study shows gender-specific differences in the utilization behavior of ED patients. According to this, women primarily visit the ED during the day and at weekends, while the proportion of male emergency patients is higher at night. This could be explained, for example, by gender-specific disease incidences and their individual occurrence. Furthermore, it is possible that women visit the ED more often at times when they see tasks of family life secured by family support or institutional support. Regarding time of admission, two new aspects should be mentioned: (1) In addition to an earlier increase in the number of visits on Monday mornings, a subsequent increase in the afternoon on Fridays was seen. It could be hypothesized that these patients were referred to the ED by general practitioners or specialists at the beginning of the week for treatment or clarification of deteriorating general health or progressive developments of chronic diseases. The late shift in the number of visits on Fridays could be explained by the fact that patients prefer to go to the ED for rapid clarification of a medical health problem before the weekend (4). These effects might also be affected by the urban location of both EDs and need to be confirmed by multicenter analyses. (2) The results also show the increased need for more and experienced clinical personnel on weekends, which hospitals in some countries are not able to meet because of collective bargaining regulations (e.g., limited shits of weekends per month) and thus could be facilitated in cooperation with SHI-physicians. This is part of the current reform efforts for emergency care in Germany, which seem essential following the results shown in this study, showing an increased use of EDs at times when outpatient care is only available to a limited extent, especially by non-urgent patients with outpatient care needs (18, 21). The aim is not only to counteract the strong sectoral separation in the German health care system through closer cooperation between outpatient and hospital-based emergency care, through the establishment of integrated emergency centers, portal practices and networks of partner practices, but also to take into account the utilization behavior and treatment needs in emergency care when planning these structures.

## Limitations

The analysis of secondary data is bound to some limitations, which must be critically reflected. The data quality of the evaluated data set was influenced by the documentation quality and documentation routine of the ED staff. The regular rotations of medical staff in the ED can lead to differences in documentation routine. Likewise, different medical specialties work in the ED with different documentation routines. Moreover, there are only few mandatory fields in the ED documentation and thus sometimes information is documented in free text fields or is only included in the physician's letter. Those data are not possible to extract for a high number of patients automatically. And last but not least another factor are the various documentation systems. In recent years, there have been many efforts to promote standardization in the documentation with slowly emerging success. Missing data occurred for gender in 0.2% of visits and for the urgency of treatment in 4.6% of visits. A high proportion of outpatient visits (74.5%) were not traced regarding mortality, but the mortality rate is expected to be low. For this reason, in-hospital mortality was determined on the basis of inpatient visits, which were completely available. In addition, the assessment of treatment urgency by MTS should be mentioned critically. The performance of the MTS by the responsible nursing staff depends on a variety of different factors, which may affect the assignment to treatment urgency levels. The assignment to the respective level of treatment urgency not only depends on the presenting leading symptom and symptom severity, it can be assumed that the utilization behavior itself has an impact on the classification of patients and thus influences the results for the assessment of treatment urgency.

Furthermore, there is currently no unique identifier for emergency treatment, thus a small proportion of patients called in for pre- or post-operative treatment in the ED might be included in the analyses (22–24). The representativeness of the sample is limited by the fact that both EDs are located in urban areas and are connected to hospitals providing maximum care. Further multi-center analyses should follow.

## Conclusion

The current study examined temporal and seasonal trends in the use of EDs with respect to demographic characteristics, treatment urgency, case type (inpatient, outpatient), and in-hospital mortality according the emergency visits by season, day of the week and holidays. The peak ED demand occurs in weekends, in spring, on holidays and school vacations (ordered by the size of effect). Those are all periods of play when people are engaged in risky behavior. Thus, hospital administrators would be wise to have low levels of staffing when most people are at work or school, and high levels when they are mostly at play. Furthermore, these results suggest that, in particular, the resource planning of outpatient emergency treatment capacities on weekends and holidays should be adapted to the increased volume of non-urgent, outpatients visits in EDs during these periods. In addition to the utilization itself, treatment urgency and local patterns of utilization should also been considered as factors in resource planning and health care measures. In particular the increased less urgent utilization at weekends and on public holidays, as well as on weekdays between 8 a.m. and 7 p.m. should be addressed by appropriate measures like increased clinical staff, on-call services by SHI-accredited doctors or additional GP services in the ED. The results of this study provide important indications for personnel and resource planning, as well as starting points for the further development of innovative, especially outpatient care structures and general practitioner cooperatives in emergency care in Germany. Based on the results, data on the urgency of patient treatment should be considered in addition to the pure case number consideration for appropriate personnel planning and the development of flanking outpatient services on weekends, school vacations and holidays. These measures could not only reduce the workload of medical staff, but also shorten waiting times for emergency patients and have a positive effect on the quality of treatment, also for more urgent cases whose outcome might be deteriorated by ED-crowding.

## Data Availability Statement

The dataset generated and analyzed during the current study is available from the corresponding author (Jennifer Hitzek) on reasonable request.

## Ethics Statement

The studies involving human participants were reviewed and approved by Charité—Universitätsmedizin Berlin. Written informed consent for participation was not required for this study in accordance with the national legislation and the institutional requirements.

## Author Contributions

JH, AS, AF-R, and MM were involved in the conception and design of the study, the acquisition, analysis and interpretation of data and approved the final version to be published, and are accountable for all aspects of the work. JH drafted the manuscript. JH and AS serve as guarantors for the manuscript. SK was involved in the interpretation of data, critically revised the manuscript for important intellectual content, approved the final version to be published and agreed to be accountable for all aspects of the work. All authors contributed to the article and approved the submitted version.

## Funding

We acknowledge support from the German Research Foundation (DFG) and the Open Access Publication Fund of Charité - Universitätsmedizin Berlin.

## Conflict of Interest

The authors declare that the research was conducted in the absence of any commercial or financial relationships that could be construed as a potential conflict of interest.

## Publisher's Note

All claims expressed in this article are solely those of the authors and do not necessarily represent those of their affiliated organizations, or those of the publisher, the editors and the reviewers. Any product that may be evaluated in this article, or claim that may be made by its manufacturer, is not guaranteed or endorsed by the publisher.

## References

[1] Berchet C. Emergency Care Services: Trends, Drivers and Interventions to Manage the Demand. Paris: OECD Health Working Papers (2015) No. 83.

[2] Zi Zentralinstitut Fue de Kassenarztliche Versorgung in deutschland. Zahlen zur ambulanten Notfallversorgung in Deustchland. Berlin (2019).

[3] Haas C L M Schöpke T Lübke-Naberhaus KD Schmidt C Brachmann M. Gutachten zur ambulanten Notfallversorgung im Krankenhaus -Fallkostenkalkulation und Strukturanalyse der Management Consult Kestermann GmbH (MCK) erstellt in Kooperation mit der Deutsche Gesellschaft interdisziplinäre Notfall- und Akutmedizin e. V. (DGINA).

[4] Dräther H M C. Ambulante Notfallversorgung an Krankenhäusern und durch ambulante Leistungserbringer. Krankenhaus-Report 2016 (2016). p. 43–62.

[5] Zimmermann M Brokmann JC Gräff I Kumle B Wilke P Gries A. Zentrale Notaufnahme – Update 2016. Anaesthesist. (2016) 65:243–249. 10.1007/s00101-016-0142-y 26952123

[6] Schmiedhofer MH Searle J Slagman A Möckel M. Inanspruchnahme zentraler Notaufnahmen: Qualitative Erhebung der Motivation von Patientinnen und Patienten mit nichtdringlichem Behandlungsbedarf. Gesundheitswesen. (2017) 79:835–44. 10.1055/s-0042-100729 27104309

[7] Searle J Muller R Slagman A Schäfer C Lindner T Somasundaram R. Überfüllung der Notaufnahmen. Notf Rett Med. (2015) 18:306–15. 10.1007/s10049-015-0011-2

[8] Somasundaram R Geissler A Leidel B Leidel BA Wrede CE. Beweggründe für die Inanspruchnahme von Notaufnahmen–Ergebnisse einer Patientenbefragung. Gesundheitswesen. (2018) 80:621–7. 10.1055/s-0042-112459 27611882

[9] Geissler A Quentin W Busse R. Umgestaltung der Notfallversorgung: Internationale Erfahrungen und Potenziale für Deutschland. Krankenhaus-Report 2017 (2017). p. 41–59.

[10] Busse Reinhard BM. Germany: health system review. Health Syst Transit. (2014) 16:1–269. 25115137

[11] Johnson KD Winkelman C. The effect of emergency department crowding on patient outcomes: a literature review. Adv Emerg Nurs J. (2011) 33:39–54. 10.1097/TME.0b013e318207e86a 21317697

[12] Carter EJ Pouch SM Larson EL. The relationship between emergency department crowding and patient outcomes: a systematic review. J Nurs Scholarsh. (2014) 46:106–15. 10.1111/jnu.12055 24354886 PMC4033834

[13] Chen YF Armoiry X Higenbottam C Cowley N Basra R Watson SI. Magnitude and modifiers of the weekend effect in hospital admissions: a systematic review and meta-analysis. BMJ Open. (2019) 9:e025764. 10.1136/bmjopen-2018-025764 31164363 PMC6561443

[14] Martinez ME. The calendar of epidemics: seasonal cycles of infectious diseases. PLoS Pathog. (2018) 14:e1007327. 10.1371/journal.ppat.1007327 30408114 PMC6224126

[15] von der Lippe P. Deskriptive Statistik. Gustav Fischer Verlag (1993). p. 397–8.

[16] Rising WR O'Daniel JA Roberts CS. Correlating weather and trauma admissions at a level I trauma center. J Trauma. (2006) 60:1096–100. 10.1097/01.ta.0000197435.82141.27 16688076

[17] McHale P Wood S Hughes K Bellis MA Demnitz U Wyke S. Who uses emergency departments inappropriately and when - a national cross-sectional study using a monitoring data system. BMC Med. (2013) 11:258. 10.1186/1741-7015-11-258 24330758 PMC3886196

[18] Scherer M Lühmann D Kazek A Hansen H Schäfer I. Patienten in Notfallambulanzen - Querschnittsstudie zur subjektiv empfundenen Behandlungsdringlichkeit und zu den Motiven, die Notfallambulanzen von Krankenhäusern aufzusuchen. Dtsch Arztebl Int. (2017) 114:645–52. 10.3238/arztebl.2017.0645 29034865 PMC5651827

[19] Trentzsch H Dodt C Gehring C Veser A Jauch KW Prückner S. Analyse der Behandlungszahlen in den Münchener Notaufnahmen des Jahres 2013/2014. Gesundheitswesen. (2019) 82:431–40. 10.1055/a-0925-8989 31394580

[20] Dormann H Diesch K Ganslandt T Hahn EG. Kennzahlen und Qualitätsindikatoren einer medizinischen Notaufnahme. Dtsch Arztebl Int. (2010) 107:261–7. 10.3238/arztebl.2010.0261 20458367 PMC2864440

[21] Slowik MWC Dräther H Fahlenbrach C Richard S. Sektorübergreifende Neuordnung der Notfallversorgung. Krankenhaus-Report 2018. (2018) p. 233–55.

[22] Augurzky B Beivers A. Rettung für die Notfallmedizin Gesundheit und Gesellschaft. (2015) 18:19–23.

[23] Wahlster P Czihal T Gibis B Henschke C. Sektorenübergreifende Entwicklungen in der Notfallversorgung–Eine umfassende Analyse ambulanter und stationärer Notfälle von 2009 bis 2015. Gesundheitswesen. (2019) 82:548–58. 10.1055/a-0820-3904 30786291

[24] Schöpke T Dodt C Brachmann M Schnieder W Petersen P-F Böer J. Statusbericht aus deutschen Notaufnahmen. Notf Rett Med. (2014) 17:660–70. 10.1007/s10049-014-1950-8

